# Dataset on Forest Bird Communities: Fragmentation Metrics & Manipulated Social Information Cues

**DOI:** 10.1038/s41597-025-06030-4

**Published:** 2025-11-05

**Authors:** Michał Bełcik, Sylwia Pustkowiak

**Affiliations:** 1 Institute of Nature Conservation, Polish Academy of Sciences, Mickiewicza 33, Kraków, 31-120 Poland; 2 Population Ecology Lab, Institute of Environmental Biology, Faculty of Biology, Adam Mickiewicz University, Uniwersytetu Poznańskiego 6, 61-614 Poznań, Poland

**Keywords:** Behavioural ecology, Forest ecology

## Abstract

Nature conservation aims to prevent species loss, often driven by habitat fragmentation. While island biogeography theory informs many models, animals consider both habitat structure but also on the social conditions in a given area when selecting territories. Individuals assess resource availability, competition, and predation risk through social cues, yet the interaction between such information (attractive vs. repulsive) and the physical properties of the habitat remains poorly understood. We provide data on both, habitat features (forest parameters and fragmentation metrics) and bird populations along with a large-scale experiment manipulating social information sources (attractive: common forest bird species, repulsive: common forest predator, mixed: attractive and repulsive alternated), testing how different local conditions scenarios affect bird populations. These data can inform broader analyses of bird responses to environmental and social factors, supporting large-scale assessments of habitat selection and population trends. Comparing effect sizes across similar studies can reveal spatiotemporal trends in bird population responses to the interaction of social and environmental cues on larger scales.

## Background & Summary

Habitat fragmentation and habitat loss are now recognised as one of the primary causes of global biodiversity decline^1–3^. Island biogeography^4^ and metapopulation theory^5^ predict that in fragmented landscapes the isolation of habitat patches can negatively affect biodiversity harboured by those patches due to a lower colonization-to-extinction ratio, leading to decreased species population density and occupancy in small, isolated habitat patches compared to larger and less isolated ones^4,5^.

Traditionally, the most commonly used index of the biodiversity within a habitat patch has been the species richness^6^. Phylogenetic diversity, reflecting life’s evolutionary heritage, and functional diversity, which influences the rate and reliability of ecosystem processes^7,8^, are another key components of biodiversity. While there is often high redundancy in functional and phylogenetic diversity in species communities^9,10^, continued species extinction inevitably leads to irreversible degradation of ecosystem functions^11^. Thus, the three above-mentioned biodiversity components may show different responses to measures of fragmentation^12–15^.

Local biodiversity may depend not only on the spatial configuration and structural features of habitat patches, but also on various intra- and interspecific interactions among individuals (e.g. social, competitive, and predator-prey interactions)^16,17^. Animals often use social public information (hereafter referred to as “social information”), such as the presence of other individuals, their sounds, pheromones, traces of their presence, their behaviour or their activities that relate to habitat quality, suitability, and available resources, while selecting and settling in a new area^16,18–20^. Individuals who utilise social information can significantly increase their fitness compared to individuals who rely solely on the structural and physical characteristics of the environment^21,22^. Social information can be carried by other individuals of the same species or different species^23^ and can affect the whole community^24,25^. Moreover, social information may originate from distinct sources that convey conflicting cues. For example, the presence of predators may create a landscape of fear, which is a continuous spatial variation in an animal’s perception of predation risk, including places where an animal avoids predation risk^26^. Therefore, the signs of presence of a predator in an area may deter prey species from setting territories in that area^27–29^ and thus decrease the level of local biodiversity^25^. In contrast, the presence of conspecifics or different species may attract individuals to settle in those habitat patches^24,27–29^.

The project, for which the data was collected, aimed to compare the response of taxonomic, phylogenetic and functional diversity of birds to forest fragmentation metrics. Another goal was to experimentally investigate the interactive effects of social information coming from two species (song thrush *Turdus viscivorus* L. and northern goshawk *Accipiter gentilis* L.) and forest fragmentation metrics (forest patch size, shape index and proximity index), on the species composition and biodiversity metrics of bird assemblages. We believe that data collected for this study may provide valuable insights on how avian communities respond to changes in soundscape, and how this influences individual birds’ decision-making processes. Published data can be used to compare responses of particular taxa, including those of conservation concern.

**Fig. 1:**
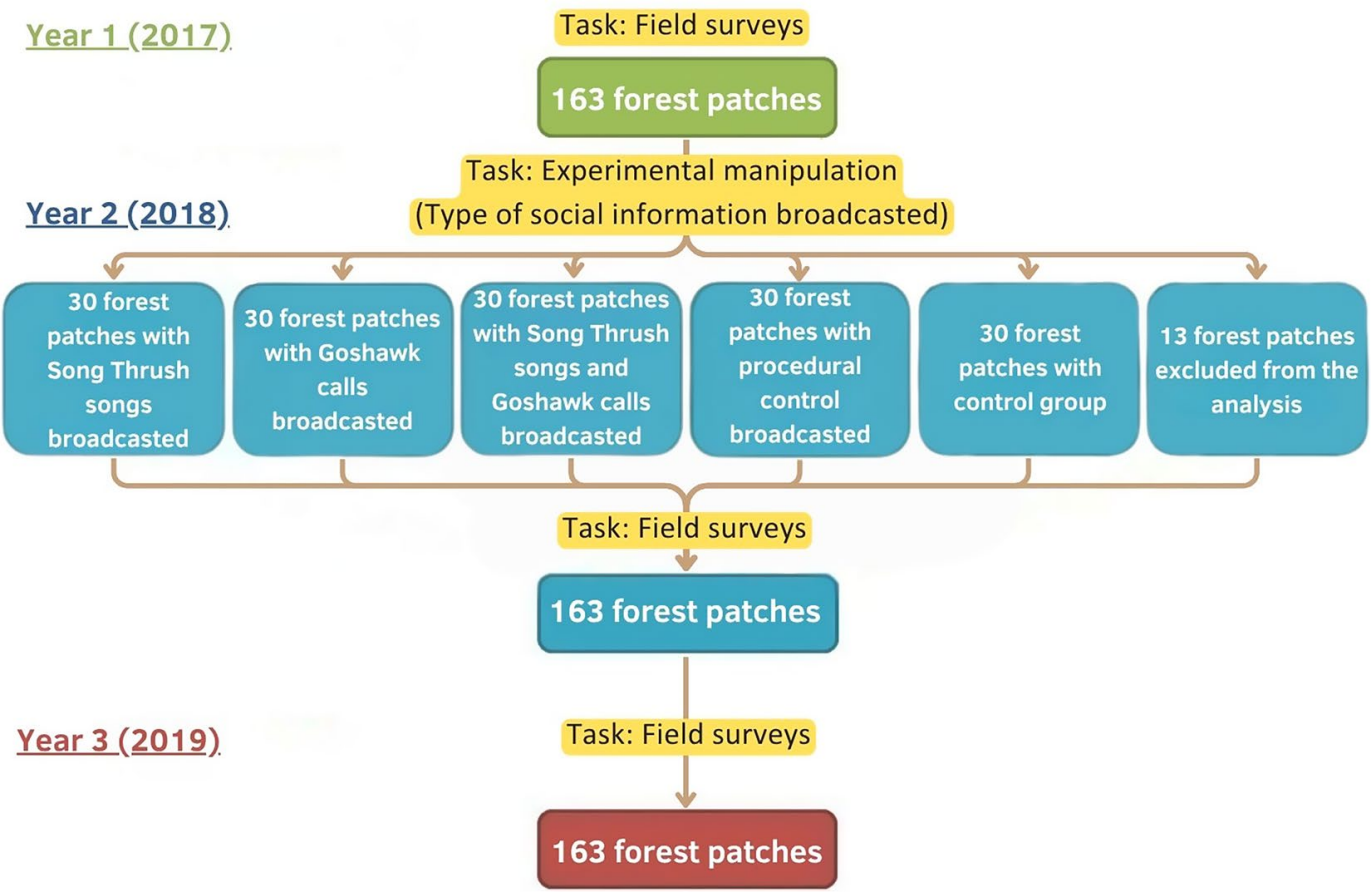
A conceptual model of the study design. Tasks for the first year of the project are shown in green, for the second year in blue, and for the third year in red. The Song Thrush songs constitute an attractive social information, Goshawk calls a repulsive social information, and the broadcast of both signals alternately constitutes mixed social information. Procedural control involves the broadcast of neutral sounds in forest patches, while control means the absence of any procedure.

For our research, conducted from 2017 to 2019, we selected 163 forest patches in the rural landscape of Southern Poland. That number exceeded the number required for experimental manipulation of social information planned for the second task, which in turn allowed us to exclude outliers based on habitat or fragmentation characteristics, ensuring comparability across experimental groups. Our project consisted of two main tasks. The first task involved field surveys to assess the avian biodiversity across all of the163 forest patches. Each patch was surveyed three times during the bird breeding season (see Methods section and Fig. 1). The second task involved a behavioural landscape experiment, where social information was manipulated in selected forest patches. During the second year, prior to field surveys, we selected five groups of 30 patches (150 patches altogether) from the initial 163 patches. Each group was then assigned different type of playback broadcast (see Methods section and Fig. 1).

## Methods

### Data overview

This dataset contains information from two sources. Data on forest patch characteristics were obtained from the Forest Data Bank (see relevant section of Methods for details). Bird diversity data were collected through field surveys performed by the birdwatchers (see relevant section of Methods for details). To provide a more detailed overview of the experimental manipulation of social information, we included descriptions of the experimental design and the construction of playback recordings used during the experiment.

### Study area

**Fig. 2:**
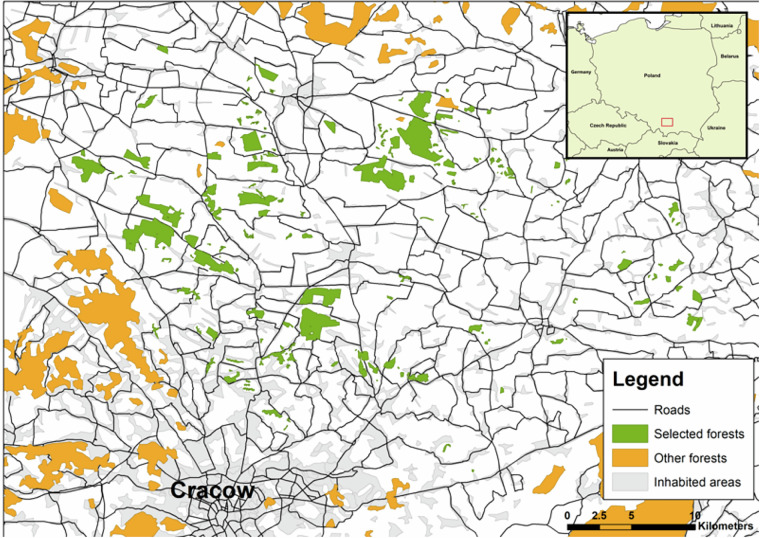
Map of the study area located north of Cracow, in southern Poland, with 163 study forest patches marked in green, and other forests marked in orange. Insert map in upper right corner indicates the location of study area in Europe (marked by the red box).

The study area, covering 1,097 square kilometres in the southern part of Poland, in the Lesser Poland Province, north of Cracow, was selected. A total of 163 forest patches located in an agricultural landscape were surveyed (Fig. 2), which constituted a significant majority of available forest patches in the area. Only the smallest patches of field boundary trees or tree clumps were not included into this selection. These patches were mainly managed by the Polish State Forests Holding and private entities (supervised by the former entity). Of note, most of these forest patches are not part of a larger continuous forest complex and differ in size and isolation. The total number of forest patches selected (163) exceeded the number of patches required by the study design for the experimental manipulation of social information, allowing for the exclusion of any outliers based on forest habitat parameters or fragmentation metrics. This approach helped ensure that experimental groups remained comparable in these variables.

### Forest patch characteristics

For majority of the forest patches, the data were obtained from the ESRI shapefiles available on the Forest Data Bank^30^ for the year 2017. These data come from the Bureau for Forest Management and Geodesy State Enterprise, the state-owned institution responsible for gathering and sharing forest data in Poland, and managing the National Forest Inventory programme. For each forest patch, a range of parameters was collected, to capture characteristics potentially important to local bird species^31^. These parameters were categorized into two groups – forest patch parameters or fragmentation metrics.

According to Bureau for Forest Management and Geodesy State Enterprise guidelines, each forest complex in Poland is divided into compartments, for which most forest stand parameters are calculated^32^. However, for this project, forest parameters needed to be calculated for entire forest patch. To achieve this, data from each forest compartment within a patch were obtained from the Forest Data Bank and averaged to represent the whole forest patch. Four forest patch parameters—forest age, share of dominant tree species, forest stand density (“compactness”), and percentage of coniferous species— were calculated this way^31^. Forest age – the mean age of dominant tree species in main stand storey (in years) – ranged 10–112 years (mean 58.18). The share of dominant tree species in main stand storey is expressed on the integer scale of 0 to 10 (with 10 being the highest result), ranged 2–10 in the presented dataset. Mean density of forest stand (“compactness”, representing percentage of forest bottom shaded by the tree canopy), ranged 30–100% (mean 66.13%). Percentage of coniferous species in main stand storey ranged 0–100% (mean 21.01%). Also, four fragmentation metrics were calculated at the forest patch level using ESRI shapefiles from the Forest Data Bank. Those were the size of the forest patch (in hectares), ranging 0.38–582.33 ha (mean 37.28 ha); nearest neighbour distance (NND, the shortest straight-line distance between a focal patch and its nearest neighbour in meters), ranging 16.53–3509.19 m (mean 269.26 m); proximity index (PROX) and Shape Index (SI)^31^. Proximity index considers the size and proximity of all neighbouring patches within a specified search radius of a focal patch. It is a sum, over all patches whose edges are within the 2.5 km radius of the focal patch, of each patch size divided by the square of its edge to edge distance from the focal patch (unitless index). In presenting data proximity index spans 0.00–1845.83 (mean 78.86). The Shape Index (SI) is a metric quantifying how complex or irregular the shape of a habitat patch is compared to a perfect square or circle. It is an unitless metric ranging from 1 (perfect circle) up with no maximum value, with higher SI means more irregular patch shape. In presented dataset SI ranges 1.110–3.528 with mean = 1.79. These metrics were computed using the Patch Analyst toolbox in ArcGis ver. 10.1, which employs the same methods for calculating landscape metrics as the Fragstats software^33^. To avoid confounding effects, patch size and isolation metrics were chosen to ensure low, non-significant correlation coefficients among them (all coefficients values below 0.2).

### Model species

In addition to analyzing the impact of forest fragmentation and social information on bird diversity metrics, we wanted to study the effects of those phenomena’s on a single model species. Our aim was also to choose a species which presence may indicate a suitable habitat, so it’s calls could be used in experimental social information broadcast. For this purpose, the song thrush (*Turdus philomelos* L.) was selected. Its songs are loud and can be heard from a distance, even outside forested areas^34^. The song thrush feeds on a variety of food sources, including both invertebrates and plant material (e.g., fruits)^35^. Moreover, it is a known prey species of the northern goshawk (*Accipiter gentilis* L.)^36^. Therefore, it’s presence may indicate a habitat rich in food resources and relatively free from predators. Our preliminary analysis, based on observations from 2017, revealed a significant positive correlation between the abundance of the song thrush and the taxonomic diversity of birds (r = 0.601, *P* < 0.001).

In experimental broadcast of social information, we also wanted to broadcast cues for the presence of a generalist predator, which may deter birds from setting territories in such forest habitat patches^37^. For that, a northern goshawk (*Accipiter gentilis* L.) was chosen. It is a large sized opportunistic predator preying upon diverse range of birds^36,38^. It breeds in various forests and even in small habitat patches, up to single trees in urban environment. Furthermore, it vocalises early during the breeding season, usually in March.

### Field surveys

Field surveys were conducted between 1^st^ of April and 31^st^ May from 2017 to 2019, with surveys in 2017 and 2019 conducted up until the 7^th^ of June. Each survey was conducted by a team of three experienced birdwatchers, each with over ten years of experience in bird censuses. Additionally, two equally experienced observers were assigned to survey forest patches due to health issues affecting the originally designated observer.”. Due to a large study area, forest patches were divided into three groups of neighbouring forests, and each observer was randomly assigned to one of these groups. Each patch was visited three times, once during each of three 20-day rounds (1^st^ -20^th^ of April, 21^st^ of April – 10^th^ of May, 11^th^-31^st^ of May). Surveys began around 5 a.m. and usually lasted until 11 a.m. During each survey, the observer recorded the starting time and moved randomly through the forest to cover as much area as possible. The observer documented every species encountered and noted the exact time the first individual of each species was heard or seen within a patch^12^. Species abundance index within a patch was estimated using the Michaelis-Menten model based on survey start time and the time of the first individual observation^39^. Every survey ended if no new species were observed for ten minutes, according to the standardized search method^40^. Mean survey duration was 59.6 minutes (SE = 21.6), with observers visiting on average 4.72 patches per day (SE = 1.18). Observers mixed the order in which they visited the patches. Before the start of each survey, the observer also recorded the wind intensity (in Beaufort’s scale), temperature (in Celsius), cloud cover (as percentage of sky covered by clouds) and rain intensity (on a categorical scale: none, light, moderate, heavy). Those variables were later included in the models.

### Experimental manipulation of social information

The field experiment was conducted from 17^th^ to 30^th^ of March 2018, just before the breeding period of the song thrush and before the start of the surveys. Prior to the experiment, forest patches were randomly assigned to one of five groups. Groups were selected to ensure no statistical differences in forest stand characteristics. The groups were as follows:

- A group of 30 patches where song thrush (*Turdus philomelos*) songs were broadcast;
- A group of 30 patches where northern goshawk (*Accipiter gentilis*) calls were broadcast;
- A group of 30 patches where both song thrush songs and goshawk calls were broadcast;
- A group of 30 patches where background noise (i.e., rustling trees, wind, and ambient landscape sounds) was broadcast as a procedural control;
- A group of 30 patches with no broadcast, serving as the control.

In each group, the assigned single broadcast type was played daily from 7 a.m. to 12 p.m. The number of loudspeakers per patch depended on its area, ranging from one to five loudspeakers, proportional to forest size to ensure complete coverage with the range of the playback broadcast. After each day’s broadcast, the speakers were collected, charged, and rehung the following morning.

### Recordings for the broadcast

Recordings of bird songs and calls with an “A” quality rating (highest quality) were obtained from the Xeno Canto portal^41^. All recordings originated from the territory of Poland (from Masovian, Lublin, Lower Silesian and Łódź Voivodeships). Original recordings were processed with a high-pass filter with cut-off at 0.5–1 kHz, depending noise level (if not already filtered in the original recording). Amplitudes was normalized across recordings to ensure consistent sound levels for each species.

**Fig. 3:**
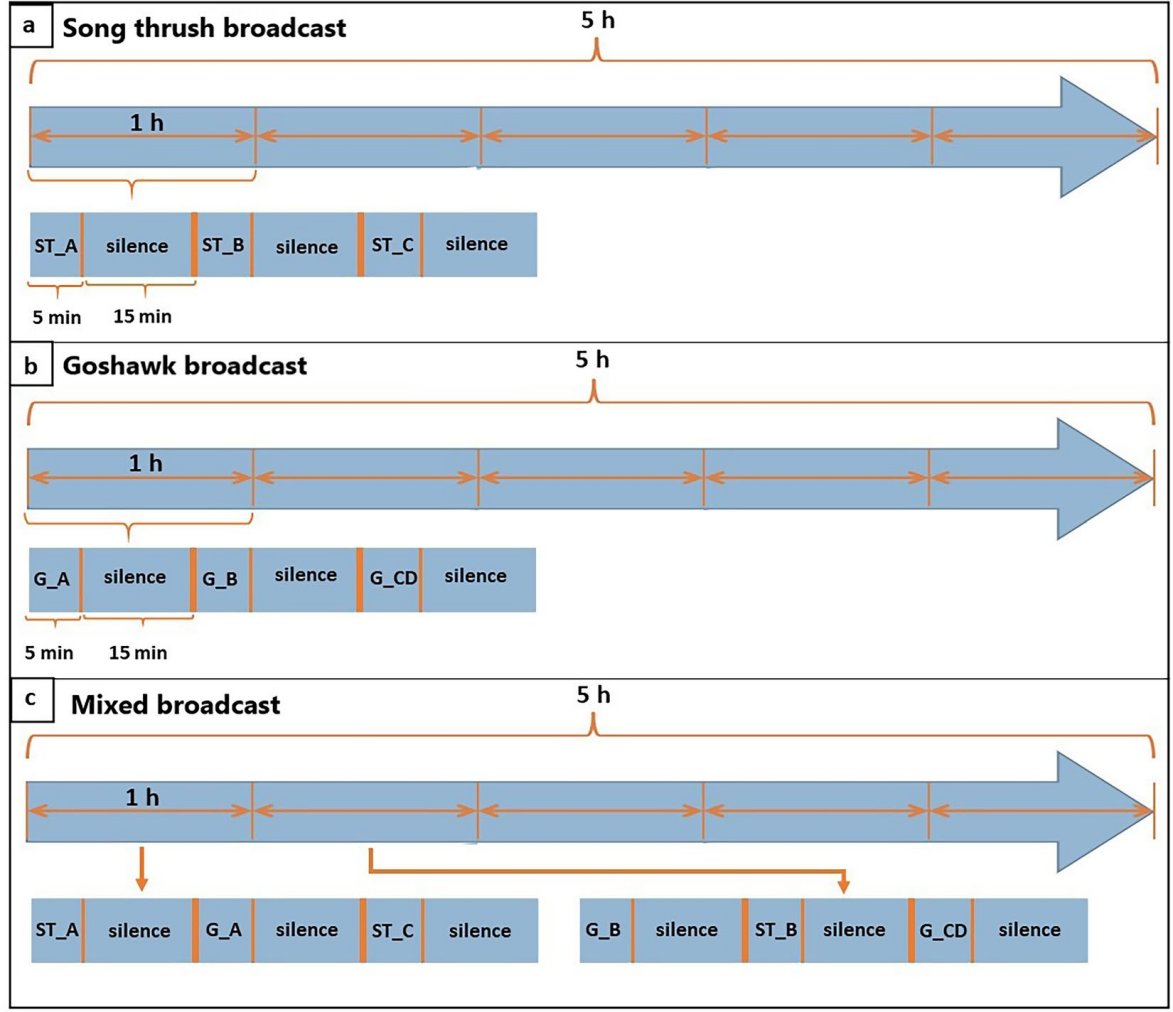
A conceptual model of the playback used in the experiment. Subgraph ‘a’ represents the playback for the song thrush *Turdus philomelos*. Subgraph ‘b’ represents the playback for the northern goshawk *Accipiter gentilis*. Subgraph ‘c’ includes vocalisations of both the song thrush and northern goshawk on the same recording. The prefix ‘ST’ denotes a sample of song thrush songs, while ‘G’ denotes a sample of northern goshawk calls. Suffixes A-D indicate sound samples from different individuals obtained from the XenoCanto, and they are further described in the Sound References section.

Each broadcast consisted of five minutes of songs/calls and fifteen minutes of silence played alternately for five hours (Fig. 3). The procedural playback was similarly constructed but featured neutral ambient sounds instead of songs or calls. The song thrush broadcast consisted of one hour-long fragment, repeated five times to create a five-hour broadcast. Each five-minute broadcast consisted of songs from a single male, with three different individuals represented in the entire playback. The broadcast of goshawk was structured similarly, except, using calls from four individuals to build the basic one-hour playback (repeated for five hours). The mixed broadcast alternated song thrush and goshawk exemplars with fifteen minutes of silence between segments, creating a two-hour fragment repeated to obtain a five-hour long broadcast (Fig. 3). As the sound samples were field recordings obtained from individuals in the real environment, they reflected natural singing/calling rates. The song thrush playback averaged 6.5 songs/minute and 81.3 syllables/minute (on average 12.4 syllables/song, min = 2, max = 65). The goshawk playback averaged 8.25 calling bouts/minute and 55.1 calls/minute (on average 6.7 calls/calling bout, min = 1, max = 23). Broadcasting devices included a JAM HX-P710 speaker set to maximum volume, paired with a Philips GoGear Azure SA5AZU08KF MP4 player. The average maximum amplitude of the song thrush songs in the playback used was 66.7 dBA, and for the goshawk calls, 62.7 dBA, measured at 1 m from the speaker with an Abatronic AB-8852 sound level meter.

## Data Records

The dataset for this project is stored on Knowledge Network for Biocomplexity^31^, under the CC0 license. The dataset for this project consists of four elements. The first element is a data table containing the forest habitat parameters and fragmentation variables for each of the forest patches, where the surveys and experimental manipulation of social information were performed (file name “Data_table_forest_parameters.xslx”). The second element is a data table containing the results of the bird surveys in the forest patches for the years 2017–2019 (file name “Data_table_bird_surveys.xslx”). Species names are consistent with the eBird webpage^42^. The third element of this dataset is an R code file (file name “forest_parameters.R”), containing code used for validating and handling the dataset “ Data_table_forest_parameters.xslx “. The fourth element of this dataset is an R code file (file name: bird_survey.R), containing code used for validating and handling the dataset “ Data_table_bird_surveys.xslx “. The description for all of the columns of both tables, as well as general description for all of the files, are included in the metadata^31^.

## Technical Validation

### Validation of experimental procedure

In the experimental manipulation of social information, in addition to the control group (lack of any manipulation), procedural control was used. The aim of this control group was to check for any unplanned effect of the experimental procedure itself on birds’ behaviour (for instance, the presence of field staff, and loudspeakers placed in the area). This was done to ensure that the result of the experiment can be interpreted as the effect of a particular broadcast.

Differences in forest parameters and fragmentation metrics among experimental groups were tested, in most cases, using ANOVA (after data transformation, if necessary). The Kruskal-Wallis test was performed for ‘Perc_coniferous’ and ‘PROX’ because the transformed data exhibited a right-skewed distribution, violating ANOVA assumptions. It was also used for ‘Compactness’ due to its categorical scale. Among most variables, we did not observe any significant differences in parameters between groups, except for the shape index (SI), where significant differences were observed between song thrush broadcast and mixed broadcast group (Table 1).

**Table 1:** Summary statistics of forest patch characteristics in particular experimental groups with the results of ANOVA analysis or Kruskal-Wallis test, depending on the distribution of the dependent variable.

	Mean ± SD	Goshawk	Mixed	Procedural control	Control	F(4, 145)	*p*
	Song thrush						
Age	53.5 ± 23.3	60.0 ± 22.5	51.6 ± 19.7	57.5 ± 27.3	57.1 ± 25.8	0.59	0.67
Share_dominant	5.9 ± 2.5	5.5 ± 2.1	4.9 ± 2.3	5.9 ± 1.8	5.5 ± 2.2	0.95	0.44
Area	12.8 ± 16.7	20.4 ± 26.4	11.1 ± 18.7	16.0 ± 17.7	14.3 ± 13.9	0.75	0.56
SI	1.6 ± 0.4	1.8 ± 0.4	2.0 ± 0.6	1.7 ± 0.5	1.8 ± 0.6	2.64	0.04*
NND	761.4 ± 890.2	565.5 ± 720.4	405.0 ± 602.5	445.6 ± 690.3	610.8 ± 667.0	1.98	0.10
	**Mean rank**						
	**Song thrush**	**Goshawk**	**Mixed**	**Procedural control**	**Control**	**χ**^2^ **(df = 4)**	***p***
Perc_coniferous	68.8	85.4	72.9	77.7	72.7	2.71	0.61
Compactness	73.7	75.8	75.4	84.7	67.9	2.92	0.57
PROX	64.8	76.3	85.5	81.8	69.1	4.70	0.32

Statistically significant effects are in bold and marked with asterisks.

### Validation of dataset integrity

Bird observations from the field surveys were initially recorded on paper forms, which were later entered into a spreadsheet by a separate team of two people. After that, data entry accuracy was verified by another team member.

**Fig. 4:**
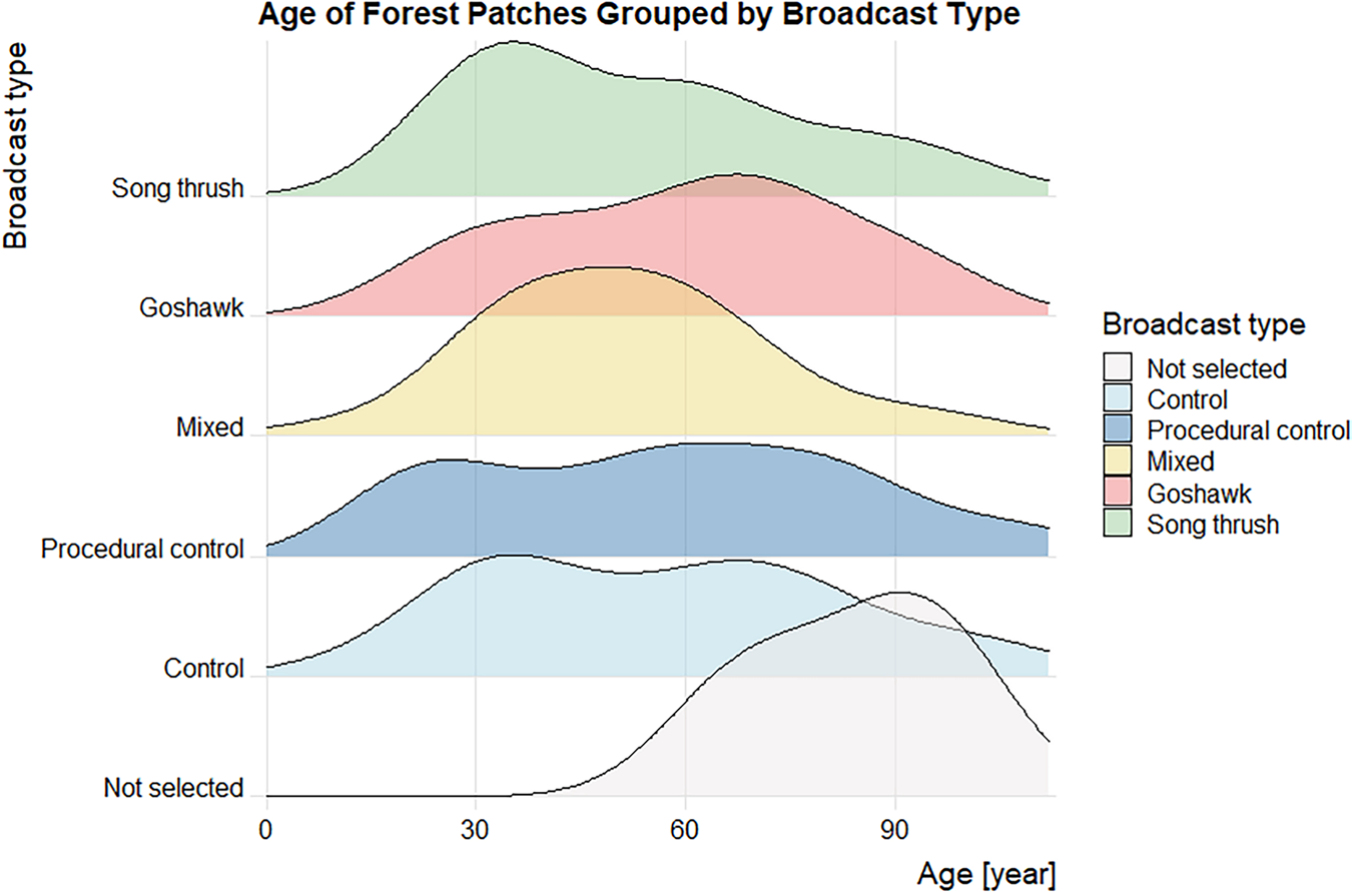
Density plot of age of forest patch, grouped by the broadcast type. Each broadcast type represents one group of forest patches, where a given type of playback was broadcasted. Group “Not selected” represent those forest patches that were not selected for the broadcast.

**Fig. 5:**
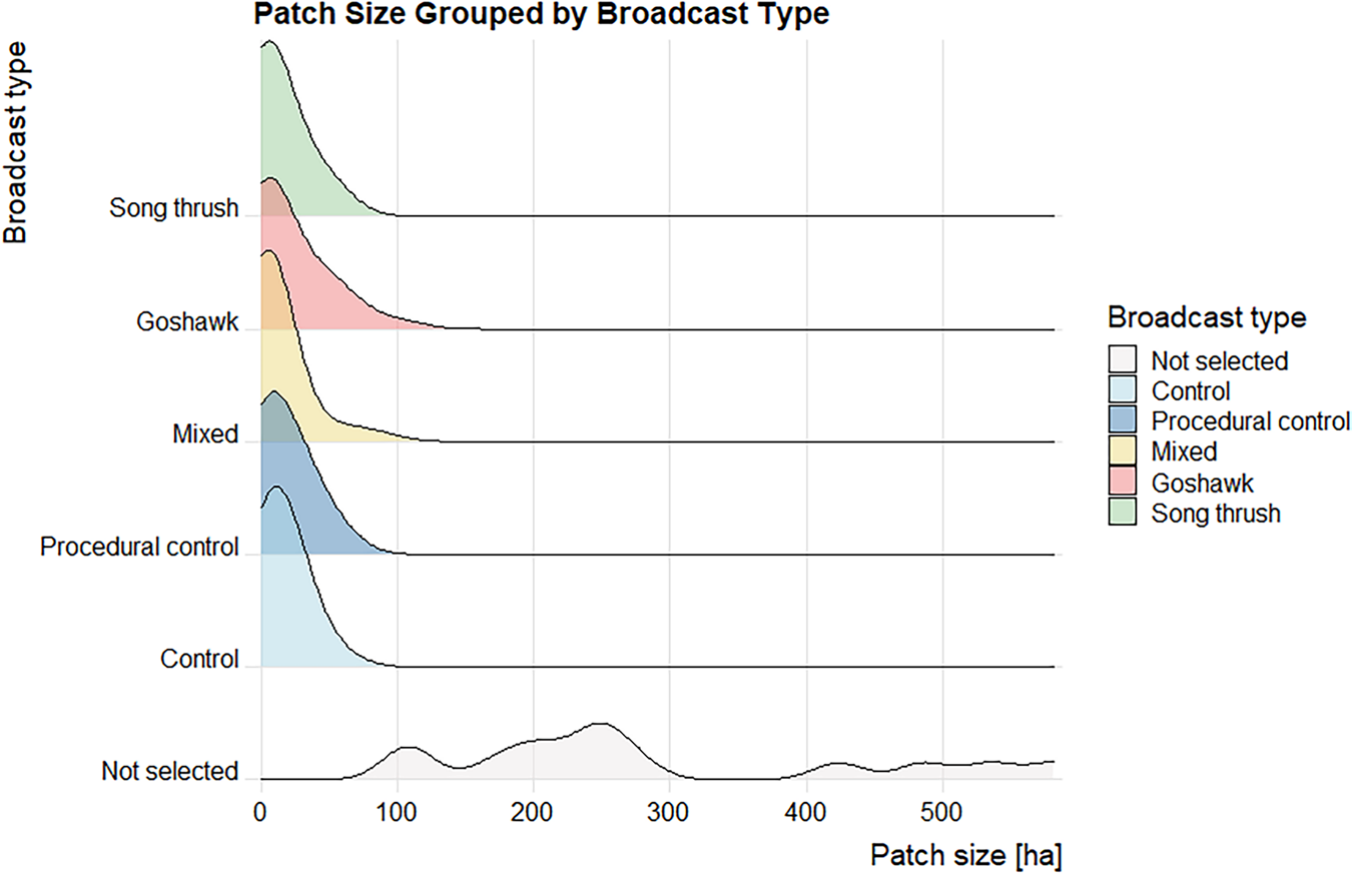
Density plot of area of forest patch, grouped by the broadcast type. Each broadcast type represents one group of forest patches, where a given type of playback was broadcasted. Group “Not selected” represent those forest patches that were not selected for the broadcast.

**Fig. 6:**
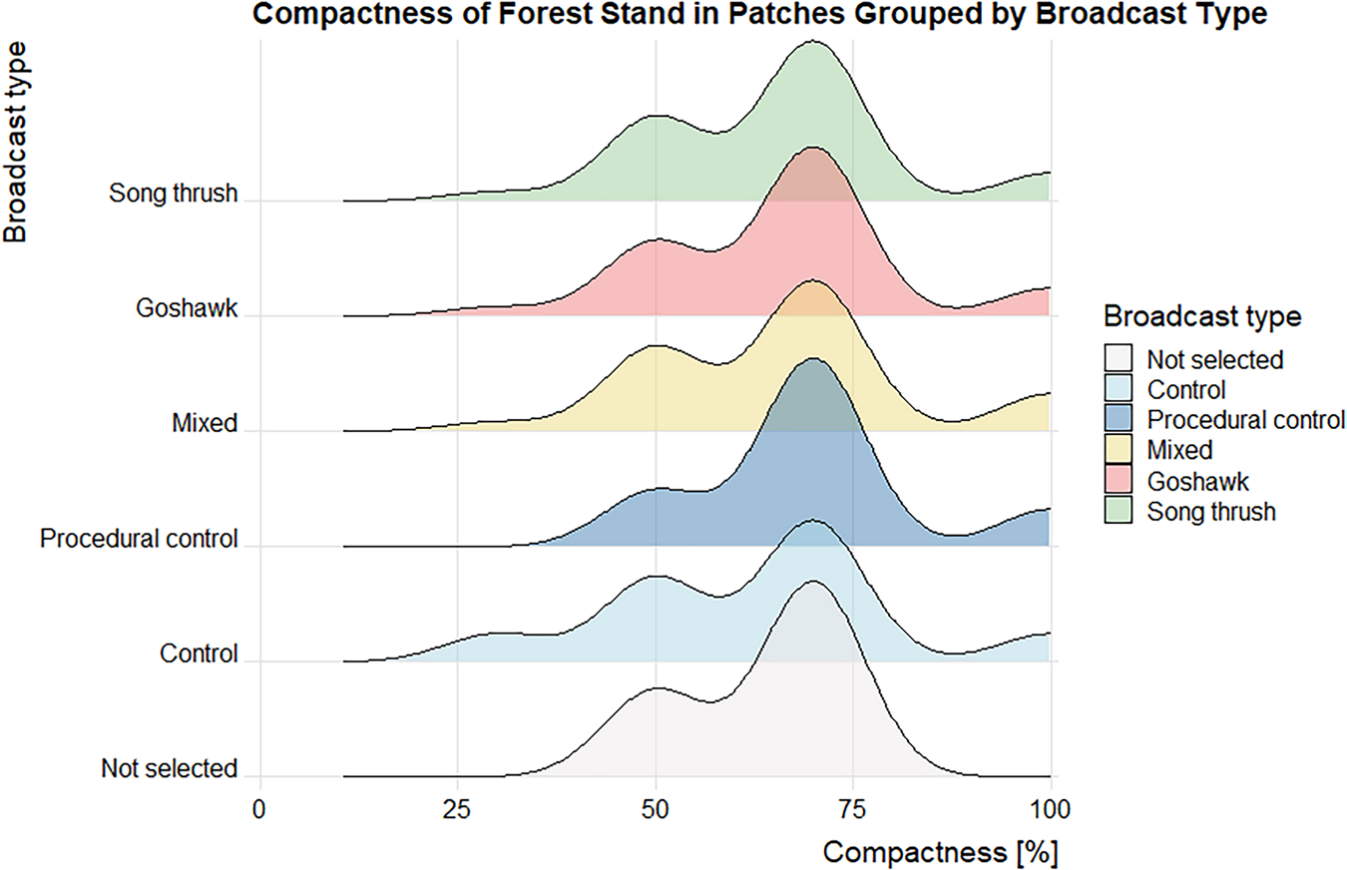
Density plot of the compactness of forest patch, grouped by the broadcast type. Each broadcast type represents one group of forest patches, where a given type of playback was broadcasted. Group “Not selected” represent those forest patches that were not selected for the broadcast.

**Fig. 7:**
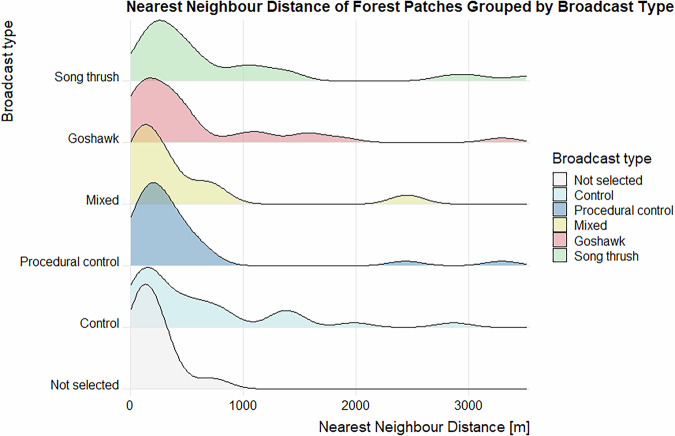
Density plot of nearest neighbour distance for each of the forest patches, grouped by the broadcast type. Each broadcast type represents one group of forest patches, where a given type of playback was broadcasted. Group “Not selected” represent those forest patches that were not selected for the broadcast.

**Fig. 8:**
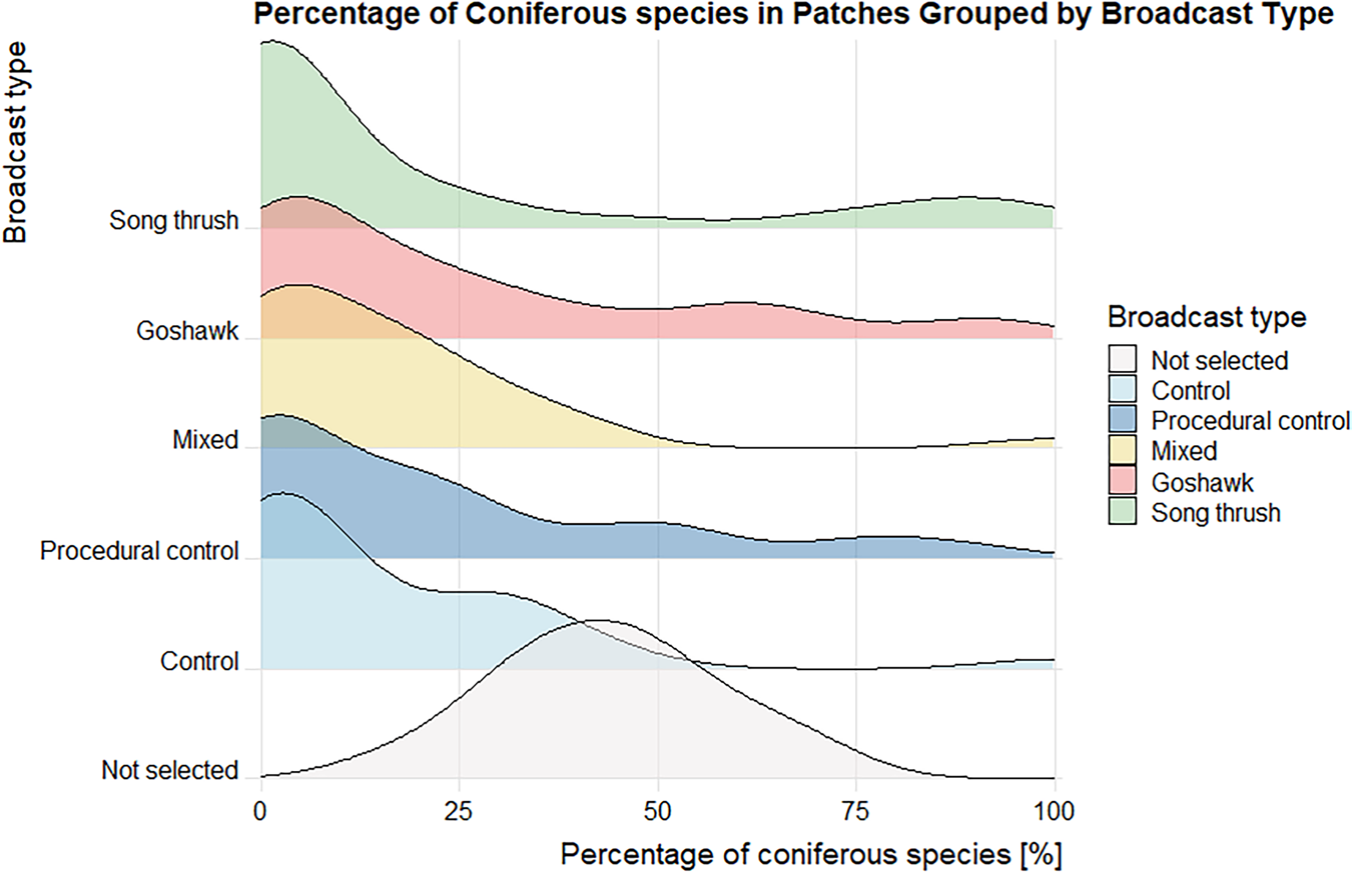
Density plot of percentage of coniferous species within forest patch, grouped by the broadcast type. Each broadcast type represents one group of forest patches, where a given type of playback was broadcasted. Group “Not selected” represent those forest patches that were not selected for the broadcast.

**Fig. 9:**
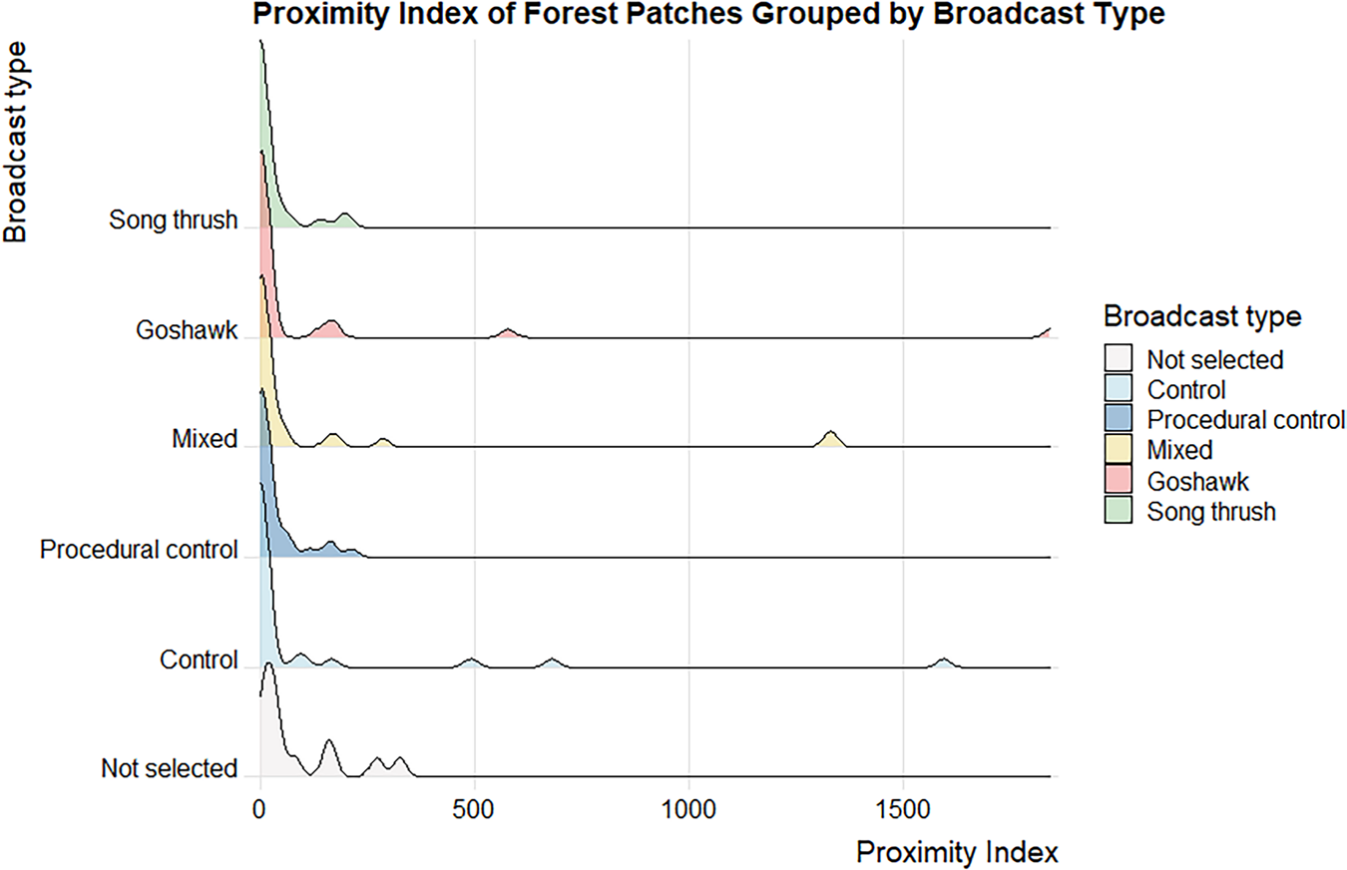
Density plot of proximity index for each of the forest patches, grouped by the broadcast type. Each broadcast type represents one group of forest patches, where a given type of playback was broadcasted. Group “Not selected” represent those forest patches that were not selected for the broadcast.

**Fig. 10:**
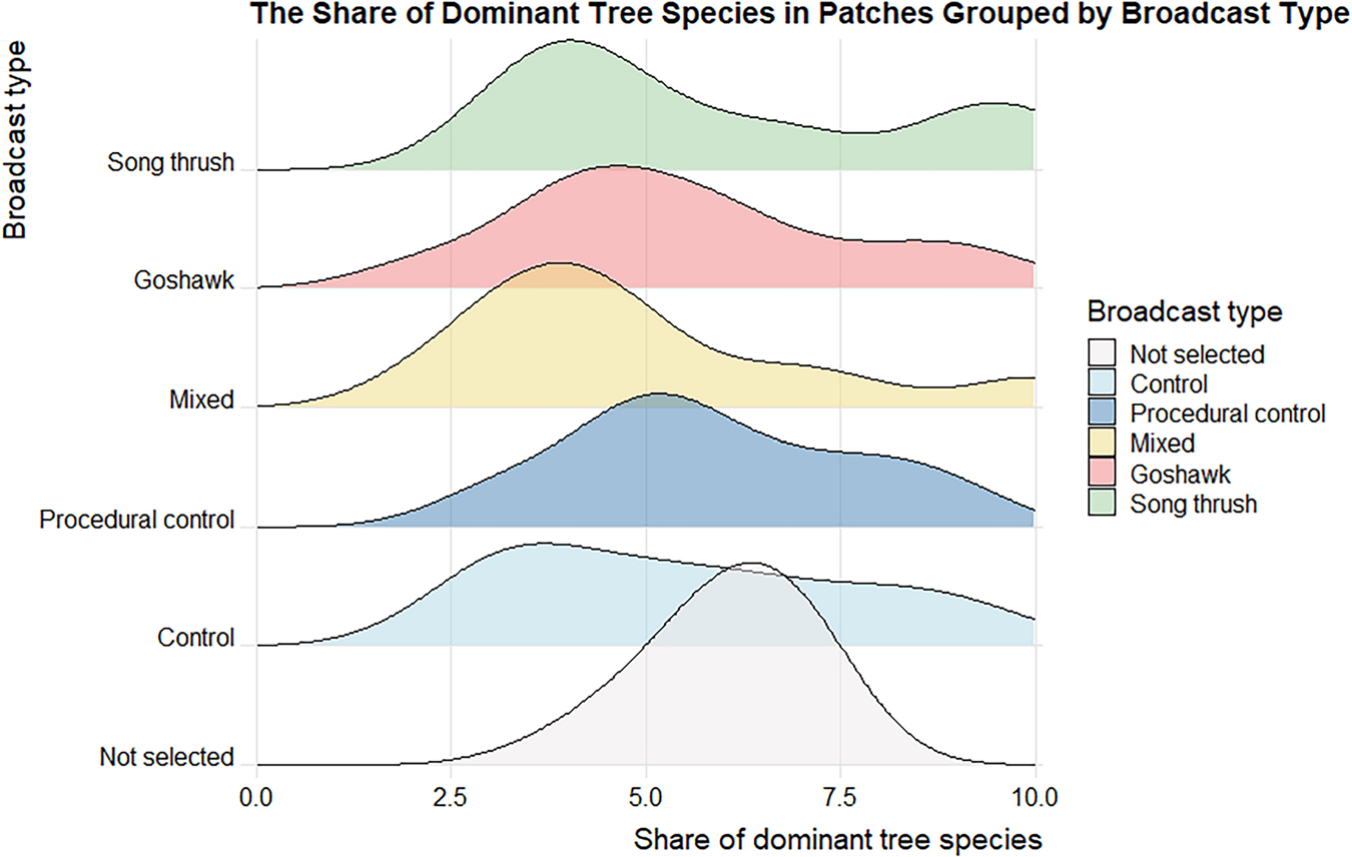
Density plot of share of dominant species within forest patch, grouped by the broadcast type. Each broadcast type represents one group of forest patches, where a given type of playback was broadcasted. Group “Not selected” represent those forest patches that were not selected for the broadcast.

**Fig. 11:**
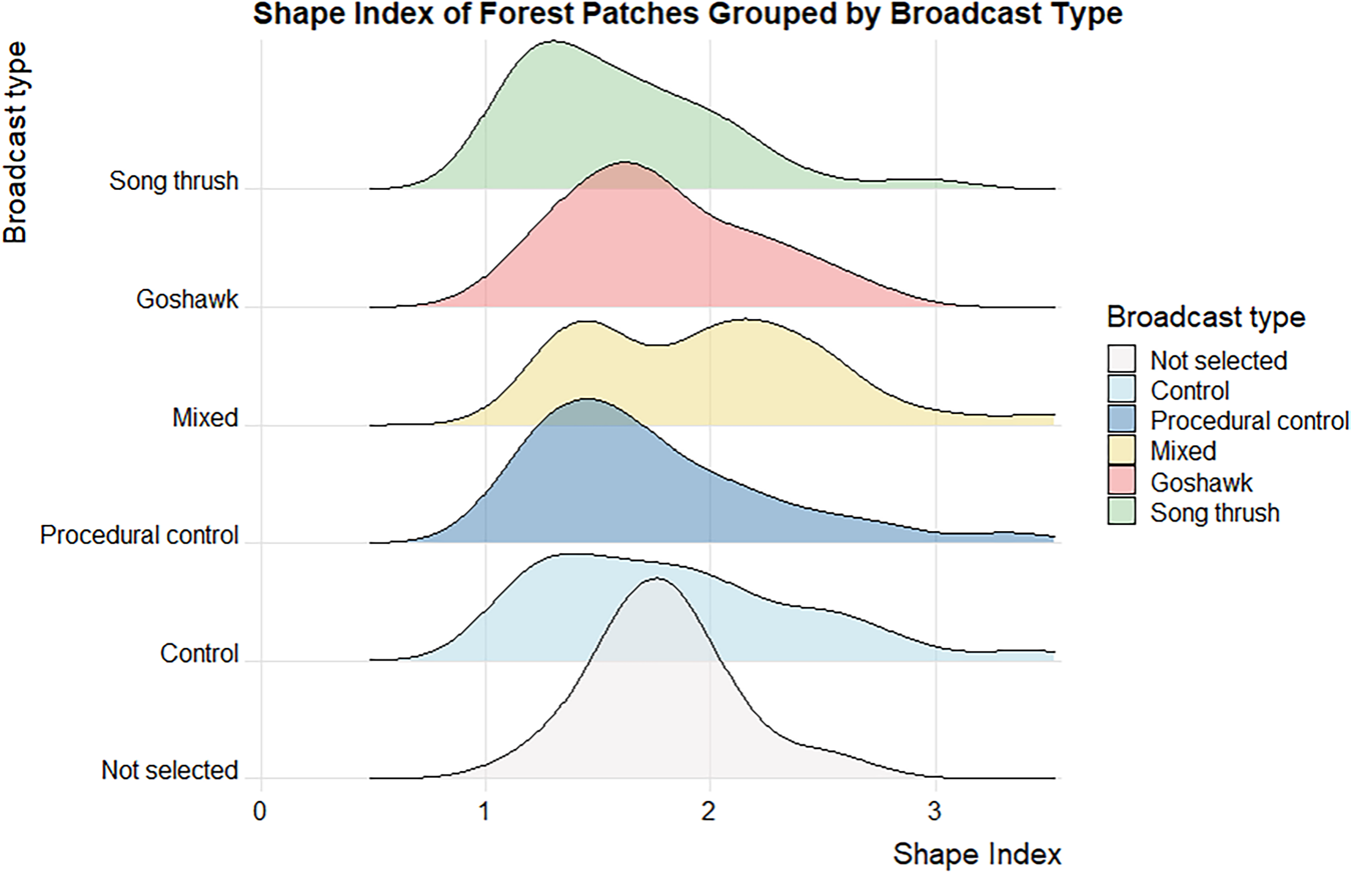
Density plot of shape index of forest patch, grouped by the broadcast type. Each broadcast type represents one group of forest patches, where a given type of playback was broadcasted. Group “Not selected” represent those forest patches that were not selected for the broadcast.

Data validation procedures wre executed in R. Using the assertion functions with specific conditions (from the “assertthat”^43^ and “assertions”^44^ packages), all the variables in the dataset on the forest parameters were checked for any missing values, duplicated rows and duplicates of the forest patch ID. Categorical variables were checked for spelling, for continuous variables, their specific value ranges were checked. The distributions of the numeric variables (forest parameters and fragmentation metrics) were presented using density plots (Figs. 4–11). The, dataset with birds’ observations was also checked for missing data and for the duplicates of a forest ID within a season and survey (Survey_ID), as well as potential duplicated surveys (duplicates among species observations). We checked categorical variables for spelling and continuous variables were checked for correct value ranges. Using data assertion techniques, we check if all of the bird observations fall within the survey period.

## Sound References

Northern goshawk A

Piotr Szczypiński 2017. *Accipiter gentilis*, XC358782. https://www.xeno-canto.org/358782.

Northern goshawk B

Dawid Jablonski 2014. *Accipiter gentilis*, XC165760. https://www.xeno-canto.org/165760.

Northern goshawk C

Piotr Szczypiński 2017. *Accipiter gentilis*, XC358611. https://www.xeno-canto.org/358611.

Northern goshawk D

Piotr Szczypiński 2017. *Accipiter gentilis*, XC358781. https://www.xeno-canto.org/358781.

Song thrush A

Antoni Knychała 2017, *Turdus philomelos*, XC367050. https://www.xeno-canto.org/367050.

Song thrush B

Antoni Knychała 2013, *Turdus philomelos*, XC138645. https://www.xeno-canto.org/138645.

Song thrush C

Joachim Rupik 2012, *Turdus philomelos*, XC105123. https://www.xeno-canto.org/105123.

## Data Availability

All the validation procedures and plots were performed using R statistical software version 4.3.2^45^. A list of all packages required to perform the analysis is at the beginning of the code. The codes used to verify the data are available in the files “forest_parameters.R” (for processing “Data_table_forest_parameters.xslx” data table) and “bird_survey.R” (for processing “Data_table_bird_surveys.xslx” data able) is available in the dataset^31^.
